# Limited value of pro-inflammatory oxylipins and cytokines as circulating biomarkers in endometriosis – a targeted ‘omics study

**DOI:** 10.1038/srep26117

**Published:** 2016-05-19

**Authors:** Yie Hou Lee, Liang Cui, Jinling Fang, Bernard Su Min Chern, Heng Hao Tan, Jerry K. Y. Chan

**Affiliations:** 1 KK Research Centre, KK Women’s and Children’s Hospital, 100 Bukit Timah Road, 229899, Singapore; 2 Singapore-MIT Alliance for Research and Technology, 1 CREATE Way, #04-13/14 Enterprise Wing, 138602, Singapore; 3 Saw Swee Hock School of Public Health, National University of Singapore, 12 Science Drive 2, #10-01, 117549, Singapore; 4 Division of Obstetrics & Gynaecology KK Women’s and Children’s Hospital, Singapore, 100 Bukit Timah Road, 229899, Singapore; 5 Department of Reproductive Medicine, KK Women’s and Children’s Hospital, Singapore, 100 Bukit Timah Road, 229899, Singapore; 6 Cancer and Stem Cell Biology, Duke-NUS Medical School, 8 College Road, 169857, Singapore.

## Abstract

Endometriosis is a common, complex gynecologic disorder characterized by the presence of endometrial-like tissues at extrauterine sites. Elevation in protein and lipid mediators of inflammation including oxylipins and cytokines within the peritoneum characterize the inflamed pelvic region and may contribute to the survival and growth of displaced endometrial tissues. The presence of a clinically silent but molecularly detectable systemic inflammation in endometriosis has been proposed. Thus, we examined serum oxylipin and immunomodulatory protein levels in 103 women undergoing laparoscopy to evaluate systematically any involvement in systemic pathophysiological inflammation in endometriosis. Oxylipin levels were similar between women with and without endometriosis. Stratification by menstrual phase or severity did not offer any difference. Women with ovarian endometriosis had significantly lower 12-HETE relative to peritoneal endometriosis (−50.7%). Serum oxylipin levels were not associated with pre-operative pain symptoms. Changes to immunomodulatory proteins were minimal, with IL-12(p70), IL-13 and VEGF significantly lower in mild endometriotic women compared to non-endometriotic women (−39%, −54% and −76% respectively). Verification using C-reactive protein as a non-specific marker of inflammation further showed similar levels between groups. The implications of our work suggest pro-inflammatory mediators in the classes studied may have potentially limited value as circulating biomarkers for endometriosis, suggesting of potentially tenuous systemic inflammation in endometriosis.

Endometriosis is a complex gynecologic disorder characterized by the presence of endometrial-like tissues at sites outside of the uterine cavity, affecting 2–10% of women, and half of women with subfertility. Pain and infertility are two prominent symptoms most commonly associated with the endometriosis and have been attributed to chronic inflammatory state of the pelvic peritoneal area with altered immunological and inflammatory milieu in the microenvironment^1^. This can be deduced by two main features found in the peritoneal environment–i) the increase in immune cells and ii) the elevation of pro-inflammatory immunomodulatory proteins (cytokines and chemokines) and lipid mediators such as prostaglandins in the peritoneum and peritoneal fluids of women with endometriosis^2^,^3^,^4^,^5^,^6^,^7^. There are several reports of increased circulating cytokines such as IL-6 and TNFα in women with endometriosis^2^,^3^,^4^,^5^ but discrepancies continue to pervade the literature in terms of reproducibility of the findings^4^,^8^,^9^. This has led to the questioning of whether endometriosis is accompanied by a clinically silent systemic inflammation^10^ and resulted in searches for circulating inflammatory markers which could potentially predict endometriosis.

Inflammation is biochemically modulated by oxylipins cooperating with cytokines and chemokines^11^. Oxylipins, collectively, includes bioactive, oxidized lipid mediators synthesized from free omega-6 polyunsaturated fatty acids (*n*-6 PUFA) including arachidonic acid (AA), linoleic acid (LA), and dihomo-gamma-linolenic acid (DGLA), or omega-3 polyunsaturated fatty acid (*n*-3 PUFA) including eicosapentenoic acid (EPA), docosahexanoic acid (DHA), and alpha-linolenic acid (ALA). Upon liberation from membrane bound phospholipids by activation of phospholipase A_2_ and subsequent oxidation by cyclooxygenase (COX), lipoxygenase (LOX), and cytochrome P450 epoxygenase (CYP450) systems, oxylipins such as prostaglandins (PG), leukotrienes (LT), thromboxanes (TBX) and hydroxyeicosatetraenoic acids (HETEs) are generated. In general, the *n*-6 PUFAs AA and LA are the precursors of pro-inflammatory lipid mediators while EPA and DHA derived lipid mediators resolve inflammation^12^. Oxylipins are known to exhibit paracrine, autocrine and increasingly endocrine effects, acting on both local and distant targets by secretion into the circulation^13^.

Considering the pivotal physiological and pathophysiological roles of oxylipins and immunomodulatory proteins in inflammation, we used them as assessors of systemic inflammation in endometriosis. We previously employed a targeted lipidomics approach using a liquid chromatography-tandem mass spectrometry (LC-MS/MS) to analyze serum sphingolipids in endometriosis^14^ and herein, we employed a combined targeted ‘omics approach comprising LC-MS/MS and multiplex immunoassay to assess the levels of serum pro-inflammatory oxylipins, cytokines and chemokines. We report limited systemic inflammation in women with endometriosis and the results was corroborated with the non-specific inflammation marker, C-reactive protein^15^. Our extensive profiling here from well-defined cases and controls suggests that systemic pro-inflammatory mediators are likely to have limited diagnostic value in endometriosis. Our results inform prospective researchers in the search for biomarker discovery of endometriosis diagnosis to refrain from pro-inflammatory mediators in the circulation.

## Methods

### Patients and sample collection

The study population comprising of 120 patients presenting with sub-fertility in addition to a general gynaecological case-mix was recruited in KK Women’s and Children’s Hospital, Singapore. Exclusion criteria include patients who are menstruating, anovulatory, post-menopausal, on hormonal therapy for at least three months before laparoscopy or anti-inflammatory medication a day before laparoscopy, and other potentially confounding diseases such as diabetes, adenomyosis or any other chronic inflammatory diseases (rheumatoid arthritis, inflammatory bowel disease, systemic sclerosis, etc). The following patients were excluded: seven menstruating patients, two with undeterminable menstrual phase, two with irregular menstrual phase, two anovulatory patients, and one patient with undetermined endometriosis severity scoring. Women provided written informed consent for collection of samples and were carried out in accordance with stipulated guidelines and regulations under Centralised Institutional Research Board approval (CIRB 2010-167-D).

A diagnostic laparoscopy was performed with careful inspection of the uterus, fallopian tubes, ovaries, pouch of Douglas and the pelvic peritoneum. Presence of endometriosis is scored according to the revised American Fertility Society (rAFS) classification of endometriosis^16^. For the purpose of this study, we grouped rAFS Stages I and II as “mild” (EM + _Mild_; *n* = 19) and rAFS Stages III and IV as “severe” (EM + _Sev_; *n* = 38). 57 patients were diagnosed as having endometriosis (EM+), and 46 women who did not have endometriosis or have benign gynecological presentations such as uterine fibroids and benign ovarian cysts were taken as the control group (EM−). Further details on patient characteristics can be found in Supplemental Table 1. Peritoneal or ovarian endometriosis (endometrioma) were determined based on endometriosis entity grouping by Chapron *et al.*^17^,^18^. There were no patients with deep infiltrating endometriosis. We estimated the sample size based on a conservative lower limit of the disease prevalence at 20% in a population and an anticipated Area Under Curve (AUC) of any candidate biomarkers of 0.8, at 90% power with Type I error (false positives) fixed at 5% and Type II error (false negatives) fixed at 5%. To this, 14 Stage I/II endometriotic subjects, 14 endometriotic Stage III/IV subjects and 56 non-endometriosis women subjects was required to power the study. A value of AUC 0.5 is of no diagnostic value and 1 representing 100% sensitivity and specificity. While slightly underpowered in terms of non-endometriosis subjects, in this exploratory study the sample size serves as a useful guide for future studies.

Menstrual cycle phase was determined according to cycle history of the patients. Blood was collected in BD Vacutainer® SST II and serum prepared by spinning the tubes at 1,200×g for 10 min and the top yellowish layer transferred to a clean 15 mL tube. Subsequently, the tube was spun at 3,600×g for 10 min. The supernatant was carefully removed and transferred into 1 mL aliquots and stored at −80 °C until use.

### Mass spectrometry analysis

The LC-MS/MS analysis followed a published report with some modifications^19^. Deuterium-labeled and non-deuterium-labeled oxylipins standards were obtained from Cayman Chemicals (MI, USA). Oxylipins were extracted from 50 μL serum by methanol–based protein precipitation and deuterated standards were added as internal standards (ISTD).

Briefly, Reversed-phase Liquid Chromatography (RPLC)-MS analysis was performed with Agilent 1290 Ultra Pressure Liquid Chromatography (UPLC, Waldbronn, Germany) coupled to an electrospray ionization with iFunnel Technology on a triple quadrupole mass spectrometer (6490 QQQ, Agilent Technologies). Chromatographic separation was achieved using HT Zorbax SB-C18 column (2.1 × 100 mm, 1.8 μm; Agilent Technologies, CA, USA) with a flow rate of 0.40 mL/min at 40 °C. The initial condition was set at 15% B, a 11 min linear gradient to 60% B was applied, followed by a 17 min gradient to 100% B which was held for 5 min, then returned to starting conditions over 0.1 min., while using Solvents A, 0.1% aqueous acetic acid, and B, 50:50 v/v acetonitrile/ isopropanol. The auto-sampler was cooled at 4 °C and 10 μL of the extract was injected. Electrospray ionization was performed in negative mode with the following source parameters: drying gas (N_2_) temperature 200 °C with a flow of 14 L/min, nebulizer gas pressure 30 psi, sheath gas temperature 400 °C with a flow of 11 L/min, capillary voltage 3,000 V and nozzle voltage 800 V. Oxylipins were quantified in Multiple Reaction Monitoring (MRM) mode and their nomenclature as defined in Supplemental Tables 2 and 3. Data acquisition and processing were performed using MassHunter software (Agilent Technologies, CA, US).

Recoveries were evaluated by spiking defined amounts of deuterated ISTDs into aliquots of unprocessed serum and calculated by comparing peak areas from serum against mean peak areas of three equal amounts of unprocessed compounds in pure solvent. The recoveries generally ranged from 55.0% to 65.2%. For intra-batch and inter-batch precision and accuracy, the relative standard deviation (RSD) values ranged from 2.5% to 18.9% and 1.5% to 15.9%, respectively. Because chromatography separated oxylipin classes according to different retention time groups, we used the closest eluting internal standard (based on structural similarity) for relative quantitation estimates. Oxylipins were quantified by normalizing to their corresponding ISTDs as described in Supplemental Table 2. Equation 1 was used for peak area normalizations for oxylipin_*i*_ of sample_*j*_ and ISTD_*k*_:


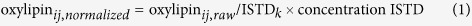


### Multiplexed immunoassay

The Luminex xMAP multiplexing technology and the Bio-Plex® platform (Bio-Rad Laboratories, CA, USA) were used as previously employed^20^. The method uses 5.5 μm polystyrene beads labelled with two fluorescent dyes in different ratios, which assigns them to specific antibodies and thus allows the simultaneous measurement of 27 immunomodulatory proteins (cytokine, chemokine and growth factor) in 25 μL of serum. Serum was diluted four times prior to analysis. Data analysis of experimental data was carried out using five-parameter logistic regression modeling on the Bio-Plex system (Bio-Rad). Calibrations and validations were performed prior to analysis and on a monthly basis respectively. Measured immuno-modulators and assay parameters are reported in Supplemental Table 4.

### C-reactive protein analysis

Serum CRP levels were determined via Architect C8000 (Abbott Diagnostics, Illinois, USA) according to manufacturer’s protocol.

### Statistics

Data were first analyzed using D'Agostino-Pearson and Shapiro-Wilk normality tests to evaluate if they followed Gaussian distribution or not. Subsequently, the appropriate tests were used to test for statistical significance–Mann Whitney and Kruskal-Wallis test for non-Gaussian distributed data and Student’s t-test and 1-way ANOVA for Gaussian distributed data. Changes were deemed significant when *p* < 0.05 and % fold change >50%.

## Results

Data from a total of 103 women who underwent diagnostic laparoscopy were used to assess changes of endometriosis-associated systemic inflammation. Of the 103 subjects, 46 women did not have endometriosis (designated “EM−”), 19 women with rAFS Stage I/II (designated “EM+_Mild_”) and 38 with Stages III/IV (designated “EM+_Sev_”) (Supplemental Table 1). The mean age was 34.6 ± 7.2 years (mean ± SD) with no statistical difference between groups. Chinese women form the majority (67.3%), followed by Malays (14.4%), Indians (7.7%) and women of other Southeast Asian heritage (10.6%). Women with proliferative or secretory phase were not significantly different between EM− and EM+. There was significant difference in the menstrual cycle comparing EM−, EM+_Mild_ and EM+_Sev_ where numbers of EM+_Mild_ women at proliferative phase were lower (*p* = 0.036). There was a significant difference of the endometriosis types between EM+_Mild_ and EM+_Sev_ (peritoneal versus ovarian endometriosis; *p* < 0.0001).

Our targeted LC-MS/MS method allowed the quantification of 50 oxylipins (validated with 50 external standards) and 5 internal standards (Supplemental Tables 2 and 3) which afforded compound identity and quantification reliability and accuracy, and also a targeted analysis of pro-inflammatory LA and AA-derived *n*-6 oxylipins. Among these 50 oxylipins, 20 were readily detectable in sera, and the four most abundant oxylipins were AA, LA, 9-HODE and 13-HODE in decreasing order (mean ± standard deviation: 68.5 ± 15.9 nM, 19.3 ± 4.2 nM, 3.3 ± 2.4 nM, 3.1 ± 2.5 nM respectively). The mean concentration levels of the remaining detectable oxylipins were <1 nM. No significant difference in serum oxylipins between EM− and EM+women was found. Stratification according to rAFS stages (I and II *versus* III and IV) or pre-operative pain symptoms did not result in significant differences relative to EM−. Stratifying by endometriosis type (ovarian/peritoneal), women with predominant endometriomas had significantly lower serum 12-HETE relative to EM− (−50.7%; *p* = 0.03) (Table 1). When matched for menstrual phase (proliferative *versus* secretory), EM− women had significantly higher 8-HETE (54.7%; *p* = 0.04), 11-HETE (61.6%, *p* = 0.02), 15-HETE (57.65, *p* = 0.03) and 5-oxoETE (52.4%; *p* = 0.04) in the proliferative phase compared to the secretory phase (Table 2). While 14,15-DHET was statistically decreased in EM+_Sev_ (*p* = 0.03), it was only 30.4% lower in the proliferative and was deemed insignificant (Table 2).

**Figure 1:**
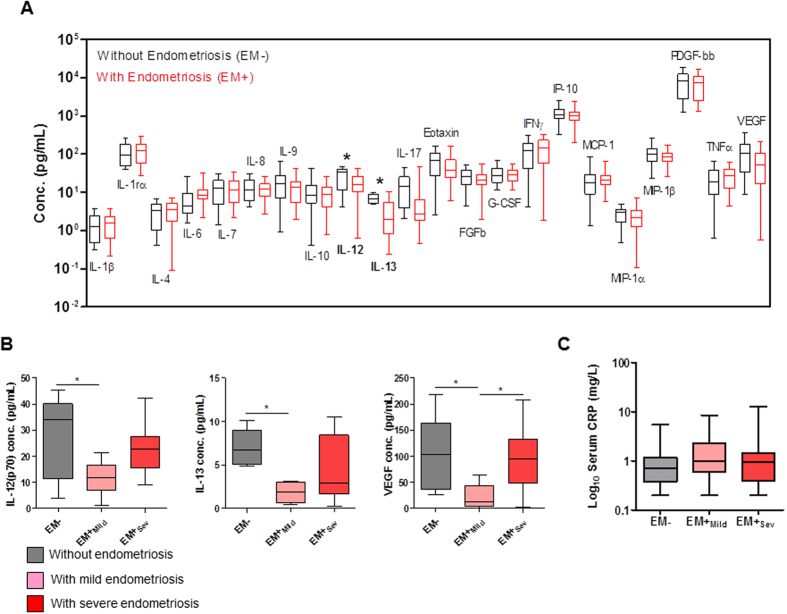
Histograms of serum immuno-modulatory proteins and C-reactive protein levels in endometriosis. (**A**) Cytokines, chemokines, growth factors, (**B**) Levels of serum IL-12(70), IL-13 and VEGF in women without endometriosis (EM−), with mild endometriosis (rAFS Stages I and II; EM+_Mild_) and with severe endometriosis (rAFS Stages I and II; EM+_Severe_). (**C**) Levels of serum C-reactive protein (CRP). 25^th^ percentile, median and 75^th^ percentile are shown by the lower, middle and upper boundaries of histogram, and whiskers show minimum and maximum. *p < 0.05

Among the 27 serum immunomodulatory proteins analyzed, 21 were detected (Table 3). The four most abundant immunomodulatory proteins were PGDF-bb, IP-10, IFNγ and IL-1rα in decreasing order (mean ± standard deviation: 7548.8 ± 4873.2 pg/mL, 1086.0 ± 501.3 pg/mL, 147.3 ± 89.2 pg/mL, 130.0 ± 70.0 pg/mL). Of the 27 factors, IL-12(p70) and IL-13 were significantly decreased by 32% and 47% between EM− and EM+ (*p* = 0.03 and 0.02 respectively; Table 2, Fig. 1A). In our study, IL-1rα, IL-6 and TNFα results were statistically insignificant between EM+to EM− (*p* = 0.90, 0.31, 0.65 respectively), EM+_Mild_ to EM− (*p* = 0.24; *p* = 0.42) and EM+_Sev_ to EM− (*p* = 0.83; *p* = 0.77). Interestingly, IL-12(p70), IL-13 and VEGF were significantly lower by 39%, 54% and 76% respectively in EM+_Mild_ compared to EM− (Fig. 1B; Table 3).

Additionally, we used serum CRP as a non-specific marker of systemic inflammation. No difference in circulating CRP levels in EM−, EM+_Mild_ and EM+_Sev_ patients was found (*p* = 0.32; Fig. 1C), with median values consistent with that of healthy volunteers^15^. CRP levels were independent of the menstrual phases (*p*_*proliferative*_ = 0.53 and *p*_*secretory*_ = 0.35).

## Discussion

Endometriosis is commonly associated with inflammation of the pelvic area and peritoneum. This hallmark has led to searches of inflammatory markers in the circulation which could potentially predict the presence of endometriosis, and the possibility of a clinically silent systemic inflammatory state in women with endometriosis^10^. Our results, which covered three classes of molecules associated with systemic inflammation, namely oxylipins, immunomodulatory proteins and CRP, were largely similar with minimal differences at a level which precludes their use as diagnostic biomarkers for endometriosis. This may explain why there has been no unequivocal consensus of the circulating cytokine levels in endometriosis^4^,^8^,^9^.

Limited changes to systemic pro-inflammatory immunomodulatory proteins and oxylipins are consistent with reports of peripheral blood immune cell activation or cytokines in women with or without endometriosis^21^,^22^. We did not find alterations in cytokines such as IL-1Rα, IL-6, TNFα as reported by other groups^2^,^23^,^24^. Similar EM+oxylipin levels in the proliferative or secretory phase is congruent with others^7^,^25^. Additionally, comparing pain symptomatic and asymptomatic groups, did not yield any significant differences between the two groups. This can be plausibly reasoned by the increased expression of neurotrophic factors and nerve fibres in endometriotic lesions, eutopic endometrium and the peritoneum, and consequently the frequent association of pain with the pelvic and uterine regions, rather than throughout the body^26^. Among the significantly different factors, with the exception of IL-13, are involved in a variety of physiologic and pathophysiologic events, and not just inflammation, such as growth factor-like (IL-12(p70), VEGF and 12-HETE). IL-12(p70) mediates anti-angiogenic effects^27^, while 12-HETE and VEGF are a potent angiogenic factors plausibly involved in the maintenance of endometriotic lesions^28^,^29^. The significant differences in immunomodulatory proteins in EM+_Mild_ compared to EM− are consistent with other reports^5^. This suggests that incremental changes in immunomodulatory proteins are likely to take place in the early phase of endometriosis development but was subverted by other unknown mechanisms later in the disease. Given the difference in our results with some reported^2^ and not others^21^,^30^,^31^ such conflicting data may be attributed to (i) the heterogeneity of the disease and/or (ii) the use of different controls in different studies: women without endometriosis but may present other benign gynecological disorders, healthy women or a combination. Alternatively, there remains a possibility that larger study cohorts may result in statistically significant findings and this study needs further verification. In addition, we did not find significant differences in serum CRP, consistent with a recent study^32^. Our results differed from another study which reported CRP levels to be significantly different in rAFS Stage III/IV endometriosis women in the first three days of the menstrual cycle^33^. Possible reasons for discrepancies include the report’s relatively smaller sample size and the temporally broader timing of sampling in our study cohort. One would note with interest that a combination of 5 proteins, permutating between plasma annexin V, VEGF, CA-125, glycodelin or sICAM-1 could predict endometriosis^34^–none of which are of pro-inflammatory nature.

Targeted ‘omics is a rapidly emerging bioanalytical field enabling the quantitative analysis of a large number of analytes associated with diseases^35^,^36^. We have previously demonstrated the elevated levels of serum sphingolipids in women with endometriosis, suggesting a different pathophysiological mechanism of these bioactive lipids to that of oxylipins in endometriosis^14^. The imbalance of *n*-3 and *n*-6 PUFAs may lead to inflammation^12^ and suggests that targeted profiling of *n*-3 PUFAs may further clarify if the role of inflammatory resolving oxylipins in endometriosis. Similarly, global ‘omics technologies including metabolomics and proteomics may further test the hypothesis of endometriosis as a systemic inflammatory disease through the potential identification of pro-inflammatory circulating metabolites or proteins. Indeed, innovation global LC-MS/MS proteomics of the serum may unravel disease-specific biomarkers^37^,^38^. Interestingly, while serum ^1^H-NMR and LC-MS/MS metabolomics of endometriosis patients revealed differential serum metabolites between endometriosis patients and those without, these metabolites were not considered of pro-inflammatory status^39^,^40^.

This study is the first to provide extensive profiles of pro-inflammatory protein and lipid mediators in the circulation of women with endometriosis and our results reflected a limited systemic inflammation in endometriosis. The implications of our work include the (i) pro-inflammatory mediators in the classes studied may have limited value as biomarkers for endometriosis, and (ii) further ‘omics work in identifying other related markers may be warranted to definitively test the hypothesis that there is systemic inflammation in endometriosis.

## Additional Information

**How to cite this article**: Lee, Y. H. *et al.* Limited value of pro-inflammatory oxylipins and cytokines as circulating biomarkers in endometriosis – a targeted ‘omics study. *Sci. Rep.* **6**, 26117; doi: 10.1038/srep26117 (2016).

## Supplementary Material

Supplementary Information

## Figures and Tables

**Table 1:** Summary of serum oxylipins in women with endometriosis (EM+) and without (EM−).

Analyte	EM− Average conc. (nM)	EM + Average conc. (nM)	EM− vs EM+ (*p*-value)	EM− vs EM+_Mild_ (*p*-value)	EM− vs EM+_Sev_ (*p*-value)	EM− vs EM+_ovar_ (*p*-value)	EM− vs EM+_perit_ (*p*-value)
5,6-DHET	0.075	0.079	0.82	0.16	0.47	0.66	0.33
8,9-DHET	0.006	0.006	0.82	0.53	0.55	0.73	0.94
11,12-DHET	0.053	0.050	0.60	0.06	0.81	0.81	0.19
14,15-DHET	0.063	0.061	0.76	0.13	0.70	0.97	0.14
12,13-DIHOME	0.693	0.632	0.58	0.67	0.65	0.44	0.86
9,10-DIHOME	0.559	0.412	0.22	0.40	0.30	0.17	0.78
5-HETE	0.061	0.060	0.91	0.95	0.85	0.83	0.77
8-HETE	0.016	0.018	0.65	0.42	0.93	0.71	0.32
11-HETE	0.227	0.201	0.66	1.00	0.54	0.66	0.91
12-HETE	0.219	0.108	0.06	0.19	0.13	0.03	0.49
13-HODE	3.041	3.211	0.74	0.90	0.59	0.45	0.86
15-HETE	0.066	0.065	0.95	0.74	0.73	0.95	0.68
20-HETE	0.004	0.004	0.47	0.08	0.94	0.91	0.20
5-HEPE	0.016	0.016	0.90	0.58	0.67	0.61	0.50
9-HODE	3.193	3.401	0.66	0.57	0.37	0.46	0.76
9,10-EODE	0.499	0.442	0.46	0.85	0.34	0.51	0.74
12,13-EODE	0.559	0.520	0.62	0.94	0.42	0.65	0.57
5-OxoETE	0.016	0.019	0.55	0.22	0.99	0.85	0.15
AA	69.094	67.976	0.72	0.62	0.85	0.71	0.49
LA	19.025	19.528	0.55	0.48	0.22	0.35	0.57

Bold, significantly changed oxylipin (% change > 50%, *p* < 0.05).

**Table 2:** Summary of serum oxylipins in women with and without endometriosis at different menstrual phases.

Analyte	% change of EM−_(Prol)_/EM−_(Secr)_	EM− Prol vs Secr (*p*-value)	% change of EM+_Mild (Prol)_/EM+_Mild (Secr)_	EM+_Mild_ Prol vs Secr (*p*-value)	% change of EM+_Sev (Prol)_/EM+_Sev (Secr)_	EM+_Sev_ Prol vs Secr (*p*-value)
9,10-DiHOME	−106.8	0.13	61.3	0.18	57.6	0.19
12,13-DiHOME	−70.3	0.07	57.3	0.12	45.4	0.13
5,6-DHET	8.1	0.55	−7.1	0.59	3.0	0.99
8,9-DHET	−3.5	0.81	−43.3	0.99	−6.5	0.55
11,12-DHET	−12.8	0.45	−34.8	0.68	−27.3	0.07
14,15-DHET	−23.0	0.19	−46.6	0.27	−30.4	**0.03**
5-HETE	34.6	0.15	−0.8	0.87	35.3	0.38
8-HETE	**54.7**	**0.04**	61.0	0.36	115.6	0.11
11-HETE	**61.6**	**0.02**	58.9	0.34	75.3	0.20
12-HETE	43.5	0.23	35.9	0.55	−43.1	0.30
13-HODE	30.4	0.14	43.7	0.42	49.9	0.20
15-HETE	**57.6**	**0.03**	62.2	0.30	80.6	0.18
20-HETE	−20.2	0.31	−8.5	0.79	−25.5	0.14
5-HEPE	18.6	0.41	−19.3	0.38	17.6	0.73
9-HODE	27.2	0.12	21.4	0.80	39.1	0.26
9,10-EODE	17.8	0.53	36.8	0.57	−15.6	0.46
12,13-EODE	3.4	0.94	40.1	0.49	−12.8	0.49
5-OxoETE	**52.4**	**0.04**	51.4	0.47	48.3	0.41
AA	5.4	0.25	−13.9	0.71	1.5	0.98
LA	2.8	0.59	−1.0	0.88	3.5	0.77

EM−, women without endometriosis; EM+_Mild_, women with rAFS I or II endometriosis; EM+_Sev_, women with rAFS III or IV endometriosis.

Prol, proliferative phase; Secr, secretory phase.

Bold, significantly changed oxylipin (% change > 50%, *p* < 0.05).

**Table 3:** Summary of serum immunomodulatory proteins in women with endometriosis (EM+) and without (EM−).

No.	Analyte	EM− Average Concentration (pg/mL)	EM+_Mild_ Average Concentration (pg/mL)	EM+_Sev_ Average Concentration (pg/mL)	% change EM+_Mild_ to EM−	% change EM+_Sev_ to EM−
1	IL-1β	1.66	1.52	1.76	8.8	−6.0
2	IL-1rα	128.29	118.58	138.18	7.6	−7.7
3	IL-4	3.51	3.33	3.90	5.3	−11.0
4	IL-6	7.54	8.12	10.87	−7.7	−44.2
5	IL-7	14.70	11.60	15.13	21.1	−2.9
6	IL-8	14.60	12.28	14.10	15.8	3.4
7	IL-9	15.79	8.51	16.36	46.1	−3.6
8	IL-10	12.60	7.63	9.94	39.5	21.1
9	IL-12 (p70)	28.66	11.74	23.33	59.0	18.6
10	IL-13	7.12	1.61	4.47	77.4	37.2
11	IL-17	17.17	2.15	9.08	87.5	47.2
12	Eotaxin	59.97	63.23	52.41	−5.4	12.6
13	FGF basic	27.38	13.23	26.87	51.7	1.9
14	G-CSF	32.28	27.82	33.85	13.8	−4.9
15	IFN-γ	144.70	131.87	158.86	8.9	−9.8
16	IP-10	1186.68	826.18	1101.85	30.4	7.1
17	MCP-1	17.90	20.21	22.55	−12.9	−26.0
18	PDGF-bb	8491.17	5277.85	7611.71	37.8	10.4
19	MIP-1β	107.28	76.52	87.59	28.7	18.4
20	TNF-α	25.25	23.03	30.69	8.8	−21.5
21	VEGF	104.87	25.43	96.96	75.7	7.5

EM−, women without endometriosis; EM+_Mild_, women with rAFS I or II endometriosis; EM + _Sev_, women with rAFS III or IV endometriosis.
