# Supplementary material for: Limited value of pro-inflammatory oxylipins and cytokines as circulating biomarkers in endometriosis – a targeted ‘omics study
Source: Sci Rep. 2016 May 19;6:26117. doi: 10.1038/srep26117 (PMC4872128; doi:10.1038/srep26117)
Supplement: Supplementary Information [file srep26117-s1.pdf]

-- Limited value of pro-inflammatory oxylipins and cytokines as circulating biomarkers in endometriosis – a targeted ‘omics study --

Yie Hou Lee[1,‡,*], Liang Cui[2,‡], Jinling Fang[3], Bernard Su Min Chern[4], Heng Hao Tan[5], Jerry K. Y. Chan[1,5,6]

[1]KK Research Centre, KK Women’s and Children’s Hospital, 100 Bukit Timah Road. Singapore 229899

[2]Singapore-MIT Alliance for Research and Technology; 1 CREATE Way, #04-13/14 Enterprise Wing; Singapore 138602

[3]Saw Swee Hock School of Public Health, National University of Singapore; 12 Science Drive 2, #10-01, Singapore 117549

[4]Division of Obstetrics & Gynaecology KK Women’s and Children’s Hospital, Singapore, 100 Bukit Timah Road. Singapore 229899

[5]Department of Reproductive Medicine, KK Women’s and Children’s Hospital, Singapore, 100 Bukit Timah Road. Singapore 229899

[6]Cancer and Stem Cell Biology, Duke-NUS Medical School. 8 College Road, Singaporei 169857

Supplemental Table 1. Study patient characteristics

	EM-	$EM+_{Mild}$	$EM+_{Sev}$	Total	*p* -value*
N	46	19	38	103	
**Average age, *years* (SD)**	36.28 (6.99)	33.79 (4.86)	35.05 (4.90)	34.6 (7.22)	0.283
**Race**					
**Chinese**	30	12	28	70	0.925
**Malay**	7	4	4	15	
**Indian**	3	1	3	8	
**Others**	6	2	3	11	
**Type**					
**Peritoneal**	NA	13	6	19	<0.0001
**Ovarian**	NA	2	36	38	
**Preoperative pain**†					
**Asymptomatic**	46	17	27	90	0.0004[a]
**Symptomatic**	0	2	11	13	0.1827[b]
**Menstrual phase**					
**Proliferative**	17	4	21	42	0.036
**Secretory**	29	15	17	61	

*1-way ANOVA was used for age; Fisher's exact or chi-square were used for the rest of the characteristics.

†Dysmenorrhea, dyspareunia, menorrhagia or pelvic pain

Table S2. Oxylipin MRM transitions and mass spectrometry parameters

No.	Analyte	Precursor Ion (m/z)	Product Ion (m/z)	Dwell	Fragmentor	Collision Energy	Cell Accelerator Voltage	Polarity	Standard type	Internal Standard
1	11,12-DHET	337.2	167.1	100	380	17	5	Negative		12,13-DIHOME-d_4
2	11,12-EET	319.2	167.1	25	380	10	5	Negative		12-HETE-d_8
3	11-HETE	319.2	167.1	25	380	12	5	Negative		12-HETE-d_8
4	11β-PGF2α	353.2	309.2	25	380	16	5	Negative		PGF2α-d_4
5	12,13-DiHOME	313.2	183.1	100	380	25	5	Negative		12,13-DIHOME-d_4
6	12,13-EODE	295.2	195.2	100	380	15	5	Negative		12,13-EODE-d_4
7	12-HETE	319.2	179.1	100	380	13	5	Negative		12-HETE-d_8
8	12-HpETE	335.2	273.3	25	380	3	5	Negative		12-HETE-d_8
9	13,14-dihydro-15-keto-PGE_2	351.2	175.2	25	380	21	5	Negative		PGF2α-d_4
10	13,14-dihydro-15-keto-$PGF_{2α}$	353.2	183.1	25	380	25	5	Negative		PGF2α-d_4
11	13-HODE	295.2	195.2	100	380	15	5	Negative		9-HODE-d_4
12	13-HpODE	311.2	183.1	25	380	36	5	Negative		9-HODE-d_4
13	14,15-DHET	337.2	207.2	100	380	17	5	Negative		12,13-DIHOME-d_4
14	14,15-EET	319.2	219.2	100	380	7	5	Negative		12-HETE-d_8
15	15-deoxy-12,14-PGD_2	333.2	271.1	25	380	12	5	Negative		PGF2α-d_4
16	15-HEPE	317.2	219.2	100	380	9	5	Negative		12-HETE-d_8
17	15-HETE	319.2	219.2	100	380	5	5	Negative		12-HETE-d_8
18	15-keto-PGE_2	349.2	287.2	25	380	11	5	Negative		PGF2α-d_4
19	15-keto-$PGF_{1α}$	353.2	317.2	25	380	8	5	Negative		PGF2α-d_4
20	1a,1b-dihomo $PGF_{2α}$	381.3	337.3	25	380	20	5	Negative		PGF2α-d_4
21	2,3-dinor-11β $PGF_{2α}$	325.2	145.1	25	380	12	5	Negative		PGF2α-d_4
22	20-HETE	319.2	289.2	100	380	17	5	Negative		12-HETE-d_8
23	20-hydroxy $PGF_{2α}$	369.2	193.1	25	380	28	5	Negative		PGF2α-d_4
24	5,15-DiHETE	335.2	173.1	25	380	10	5	Negative		12-HETE-d_8
25	5,6-DHET	337.2	145.1	25	380	14	5	Negative		12-HETE-d_8
26	5,6-DiHETE	335.2	115.1	25	380	16	5	Negative		12-HETE-d_8
27	5-HEPE	317.2	115.1	100	380	11	5	Negative		12-HETE-d_8
28	5-HETE	319.2	115.1	100	380	13	5	Negative		12-HETE-d_8

29	5-OxoETE	317.2	203.2	100	380	17	5	Negative		12-HETE-d_8
30	6,15-diketo-13,14-dihydro-$PGF_{1α}$	369.2	113.2	25	380	30	5	Negative		PGF2α-d_4
31	6-keto-PGE_1	367.2	143.1	25	380	16	5	Negative		PGF2α-d_4
32	6-keto-$PGF_{1α}$	369.2	163.1	200	380	29	5	Negative		PGF2α-d_4
33	8,9-DHET	337.2	127.1	25	380	20	5	Negative		12,13-DIHOME-d_4
34	8,9-EET	319.2	257.2	25	380	8	5	Negative		12-HETE-d_8
35	8-HETE	319.2	155.1	25	380	12	5	Negative		12-HETE-d_8
36	8S,15S-DiHETE	335.2	235.2	25	380	12	5	Negative		12-HETE-d_8
37	9,10-DiHOME	313.2	201.1	100	380	25	5	Negative		12,13-DIHOME-d_4
38	9,10-EODE	295.2	171.1	100	380	15	5	Negative		12,13-EODE-d_4
39	9-HETE	319.2	151.1	100	380	11	5	Negative		12-HETE-d_8
40	9-HODE	295.2	171.1	100	380	15	5	Negative		9-HODE-d_4
41	AA	317.2	203.2	25	380	17	5	Negative		PGF2α-d_4
42	Arachidonol ethanolamide	346.2	259.1	25	380	9	5	Negative		PGF2α-d_4
43	LA	295.2	195.2	25	380	15	5	Negative		PGF2α-d_4
44	LTB_4	335.2	195.1	25	380	12	5	Negative		PGF2α-d_4
45	LTE_4	438.2	333.2	100	380	15	5	Negative		PGF2α-d_4
46	PGA_2	333.2	271.2	25	380	12	5	Negative		PGF2α-d_4
47	PGB_2	333.2	175.1	25	380	16	5	Negative		PGF2α-d_4
48	PGE_2	351.2	271.2	25	380	14	5	Negative		PGF2α-d_4
49	PGE_2 ethanol amide	299.2	198.2	25	380	15	5	Negative		PGF2α-d_4
50	$PGF_{1α}$	355.2	293.2	25	380	21	5	Negative		PGF2α-d_4
51	$PGF_{2α}$	353.2	309.2	25	380	15	5	Negative		PGF2α-d_4
52	PGK_2	349.2	287.3	25	380	16	5	Negative		PGF2α-d_4
53	TXB	369.2	169.1	200	380	17	5	Negative		TXB-d_4
54	TXB-d_4	373.2	173.1	200	380	20	5	Negative		
55	$PGF_{2α}$-d_4	357.3	313.2	200	380	20	5	Negative		
56	9-HODE-d_4	299.2	172.1	100	380	15	5	Negative		
57	12-HETE-d_8	327.3	184.2	100	380	13	5	Negative		
58	12,13-EODE-d_4	299.2	198.2	100	380	15	5	Negative		
59	12,13-DiHOME-d_4	317.3	185.2	100	380	25	5	Negative		

Deuterated standards
With external standards
Without external standards

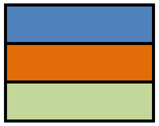

Table S3. Oxylipin nomenclature

Acronym	Full name
20-hydroxy $PGF_{2\alpha}$	20-hydroxy-prostaglandin $F_{2\alpha}$
6-keto-$PGF_{1\alpha}$	6-keto-prostaglandin $F_{1\alpha}$
2,3-dinor-11β $PGF_{2\alpha}$	2,3-dinor-11-beta-prostaglandin $F_{2\alpha}$
6-keto-PGE_1	6,15-diketo-13,14-dihydro--prostaglandin E_1
6,15-diketo-13,14-dihydro-$PGF_{1\alpha}$	6-keto-prostaglandin $F_{1\alpha}$
TXB	thromboxane B
11β-PGF2α	11-beta-prostaglandin F_2a
$PGF_{2\alpha}$	prostaglandin $F_{2\alpha}$
$PGF_{1\alpha}$	prostaglandin $F_{1\alpha}$
PGE_2	prostaglandin E_2
PGE_2 ethanol amide	prostaglandin E_2 ethanol amide
PGK_2	prostaglandin K_2
15-keto-PGE_2	15-keto-prostaglandin E_2
15-keto-$PGF_{1\alpha}$	15-keto-prostaglandin $F_{1\alpha}$
13,14-dihydro-15-keto-PGE_2	13,14-dihydro-keto-prostaglandin E_2
13,14-dihydro-15-keto-$PGF_{2\alpha}$	13,14-dihydro-keto-prostaglandin $F_{2\alpha}$
1a,1b-dihomo $PGF_{2\alpha}$	1a,1b-dihomo-prostaglandin $F_{2\alpha}$
15-deoxy-12,14-PGD_2	15-deoxy-12,14-prostaglandin D_2
PGB_2	prostaglandin B_2
LTE_4	leukotriene E_4
8S,15S-DiHETE	8S,15S-dihydroxy-eicosatetraenoic acid
5,15-DiHETE	5,15-dihydroxy-eicosatetraenoic acid
PGA_2	prostaglandin A_2
LTB_4	leukotriene B_4
12,13-DiHOME	12,13-dihydroxy-octadecenoic acid
9,10-DiHOME	9,10-dihydroxy-octadecenoic acid
14,15-DHET	14,15-dihydroxy-eicosatrienoic acid
11,12-DHET	11,12-dihydroxy-eicosatrienoic acid
8,9-DHET	8,9-dihydroxy-eicosatrienoic acid
5,6-DiHETE	5,6-dihydroxy-eicosatetraenoic acid
15-HEPE	15-hydroxy-eicosapentaenoic acid
20-HETE	20-hydroxy-eicosatetraenoic acid
5,6-DHET	5,6-dihydroxy-eicosatrienoic acid
5-HEPE	5-hydroxy-eicosapentaenoic acid
13-HODE	13-hydroxy-octadecadienoic acid
9-HODE	9-hydroxy-octadecadienoic acid
15-HETE	15-hydroxy-eicosatetraenoic acid
11-HETE	11-hydroxy-eicosatetraenoic acid
8-HETE	8-hydroxy-eicosatetraenoic acid
12-HETE	12-hydroxy-eicosatetraenoic acid
12-HpETE	12-Hydroperoxy-eicosatetraenoic acid

9-HETE	9-hydroxy-eicosatetraenoic acid
5-HETE	5-hydroxy-eicosatetraenoic acid
12,13-EODE	12,13-epoxyoctadecenoic acid
14,15-EET	14,15-epoxyeicosatrienoic acid
9,10-EODE	9,10-epoxyoctadecenoic acid
11,12-EET	11,12-epoxyeicosatrienoic acid
5-OxoETE	5-oxo-eicosatetraenoic acid
8,9-EET	8,9-epoxyeicosatrienoic acid
13-HpODE	13-hydroperoxy-octadecadienoic acid
Arachidonol ethanolamide	Arachidonol ethanolamide
AA	Arachidonic acid
LA	Linoleic acid

Table S4. Luminex assay parameters

No.	Analyte	% detected in patients	Average Intra-CV%	Working sensitivity (LOQ; pg/mL)	Assay sensitivity (LOD*; pg/mL)
1	IL-1β	100.0	6.14	2.186	0.021
2	IL-1rα	100.0	7.39	1.989	2.695
3	IL-2	5.0	6.36	0.974	0.829
4	IL-4	97.5	6.23	0.222	0.0009
5	IL-5	5.0	8.56	2.19	0.105
6	IL-6	85.0	5.92	1.205	0.0161
7	IL-7	100.0	6.59	2.361	0.141
8	IL-8	100.0	5.44	1.877	0.267
9	IL-9	70.0	4.87	2.624	0.0091
10	IL-10	77.5	6.56	1.772	0.0041
11	IL-12	85.0	10.00	2.19	0.0062
12	IL-13	50.0	5.69	2.117	0.0024
13	IL-15	5.0	7.94	1.639	2.963
14	IL-17	50.0	5.91	1.213	0.0045
15	Eotaxin	82.5	10.48	1.538	0.00261
16	FGF basic	85.0	5.25	1.064	0.0201
17	G-CSF	100.0	6.96	1.732	1.153
18	GM-CSF	35.0	5.86	0.651	0.044
19	IFN-γ	100.0	5.06	6.42	0.183
20	IP-10	100.0	5.71	8.522	20.441
21	MCP-1	95.0	5.01	1.524	0.0131
22	MIP-1α	52.5	5.73	1.409	0.00011
23	PDGF-bb	100.0	3.48	6.655	123.941
24	MIP-1β	100.0	5.29	1.395	2.307
25	RANTES	40.0	2.98	1.924	462.07
26	TNF-α	92.5	6.91	4.612	0.0062
27	VEGF	95.0	5.39	2.407	0.0056

[grey box] Not detected

* 1/10 of lowest detectable value
